# Visual bibliometric analysis of electroacupuncture research in stroke treatment: a 20-year overview

**DOI:** 10.3389/fnins.2023.1265854

**Published:** 2023-10-10

**Authors:** Hyonjun Chun, Woo-Chul Shin, Jong-min Kim, Hyungsuk Kim, Jae-Heung Cho, Mi-Yeon Song, Won-Seok Chung

**Affiliations:** ^1^Department of Clinical Korean Medicine, Graduate School, Kyung Hee University, Seoul, Republic of Korea; ^2^Department of Korean Rehabilitation Medicine, Dong-shin Korean Medicine Hospital, Seoul, Republic of Korea; ^3^Department of Korean Rehabilitation Medicine, Kyung Hee University Medical Center, Seoul, Republic of Korea; ^4^Department of Oriental Neuropsychiatry, Dong-Seo Medical Center, Seoul, Republic of Korea

**Keywords:** stroke, electroacupuncture, bibliometric analysis, Web of Science, VOSviewer

## Abstract

**Background:**

Electroacupuncture has been used as a treatment; however, a visual bibliometric analysis has not yet been performed in this field. In this study, we aimed to suggest future research topics and directions related to the field by examining the last 20 years of research trends and hotspots of electroacupuncture in stroke.

**Methods:**

We searched the Web of Science database on electroacupuncture as a treatment for stroke published from 2003 to 2022. We analyzed the papers by annual publication, research fields, nations, affiliations, authors, journals, and keywords. VOSviewer software was used to visualize the bibliometric analysis and results. A total of 440 papers were included in the analysis.

**Results:**

The number of publications has gradually increased every year, and neuroscience has become the most actively studied field. Neural Regeneration Research journal and China had the most publications. Fujian University of Traditional Chinese Medicine, as an affiliated institute, published the most articles. Chen Lidian and Tao Jing presented the largest number of papers, making them the leading contributors in this field. Four clusters were created by analyzing keywords, such as “neuroprotection,” “clinical rehabilitation,” “neuroplasticity,” and “pretreatment-induced tolerance”.

**Conclusion:**

This study is the first to analyze the research trends in electroacupuncture as a treatment for stroke using the VOSviewer. It shows the current state of research in the field by visualizing research trends and hotspots. This will help offer reference data for future studies.

## 1. Introduction

Stroke is classified into several types including ischemic stroke, subarachnoid hemorrhage, and intracerebral hemorrhage. Despite recent improvements in medicine, stroke is one of the leading causes of mortality worldwide (Feigin et al., 2021). Additionally, lengthy sequelae of stroke are frequent, contributing to an increase in social costs (Tsao et al., 2022) by decreasing the quality of life. Stroke is generally categorized into three stages; acute, sub-acute and chronic stage depending on pathological characteristics and post-stroke period (Zhao and Willing, 2018). Acute stage of ischemic stroke is treated with venous thrombolytics injection or thrombectomy whereas hemorrhagic stroke is treated with controlling hemorrhage and removing hematoma. Since majority of people who have suffered a stroke report significant disability even after appropriate acute treatment, rehabilitation treatment plays a critical role in subacute and chronic stage. Conventionally physical therapy, occupational therapy and speech therapy has been recommended for rehabilitation and management of cardiovascular risk factors such as lowering blood pressure is general principles to prevent secondary stroke (Campbell and Khatri, 2020). Despite these conventional medical care, establishment of the optimal rehabilitation intervention has been challenging. Therefore, alternative treatments such as acupuncture, electro-acupuncture and herbal medicine has been gaining attention as a rehabilitation treatment for chronic stage patients.

Traditionally in East Asia, Traditional Chinese Medicine (TCM) has been used for stroke care (Ceniceros and Brown, 1998 Zhang et al., 2022). In the early 19th century, Berlioz from France suggested the clinical application of concurrent use of modern electric therapy and traditional acupuncture and Electroacupuncture (EA) has been used in clinical environment to treat stroke and related disorders since (Macdonald, 1993). Unlike other modality of TCM, EA has the advantage of being standardized by controlling the frequency and intensity of stimulation and has been researched diversely on mechanism and its clinical effect. In animal experiments, EA alleviates ischemic brain damage by regulating apoptosis, inflammation, autophagocytosis, glutamate, mRNA, and other factors (Xing et al., 2018). In clinical applications, EA is an effective treatment for stroke symptoms such as dysphagia (Huang et al., 2020), pain (Xu et al., 2020), aphasia (Shi et al., 2022), and urinary incontinence (Cruz et al., 2022).

The bibliometric analysis method uses mathematical and statistical tools, thereby providing an overview of academic research publications and a quantitative analysis of research trends. This method can identify influential publications, authors, journals, affiliations, and countries and analyze and present topics, methods, and influential keywords (van Eck and Waltman, 2010). There are bibliometric analyses of EA regarding general diseases from 2011 to 2020 (Wei et al., 2022); however, a bibliometric analysis of EA as a treatment for stroke has not yet been conducted. Therefore, in this study, we conducted a bibliometric analysis by year, country, journal, keyword, affiliation, and author of research papers about EA for stroke, which were published in the Web of Science Core Collection Database (WOSCC) in the last 20 years. Network properties between studies and research trends were identified. Therefore, we aimed to propose additional future research directions in the field.

## 2. Methods

### 2.1. Data search

All data were extracted from WOSCC. Research papers published between January 01, 2003, and December 31, 2022, were collected, and data was extracted from articles published between February 20, 2023, and February 27, 2023, based on WOSCC using TS: ([“stroke” OR “infarction” OR “cerebrovascular accident” OR “cerebral hemorrhage” OR “cerebral ischemia”] AND [“Electroacupuncture” OR “electro-acupuncture” OR “electric acupuncture”]) as search queries. The results were then converted into text files and organized for further data analysis on March 01, 2023.

**Figure 1:**
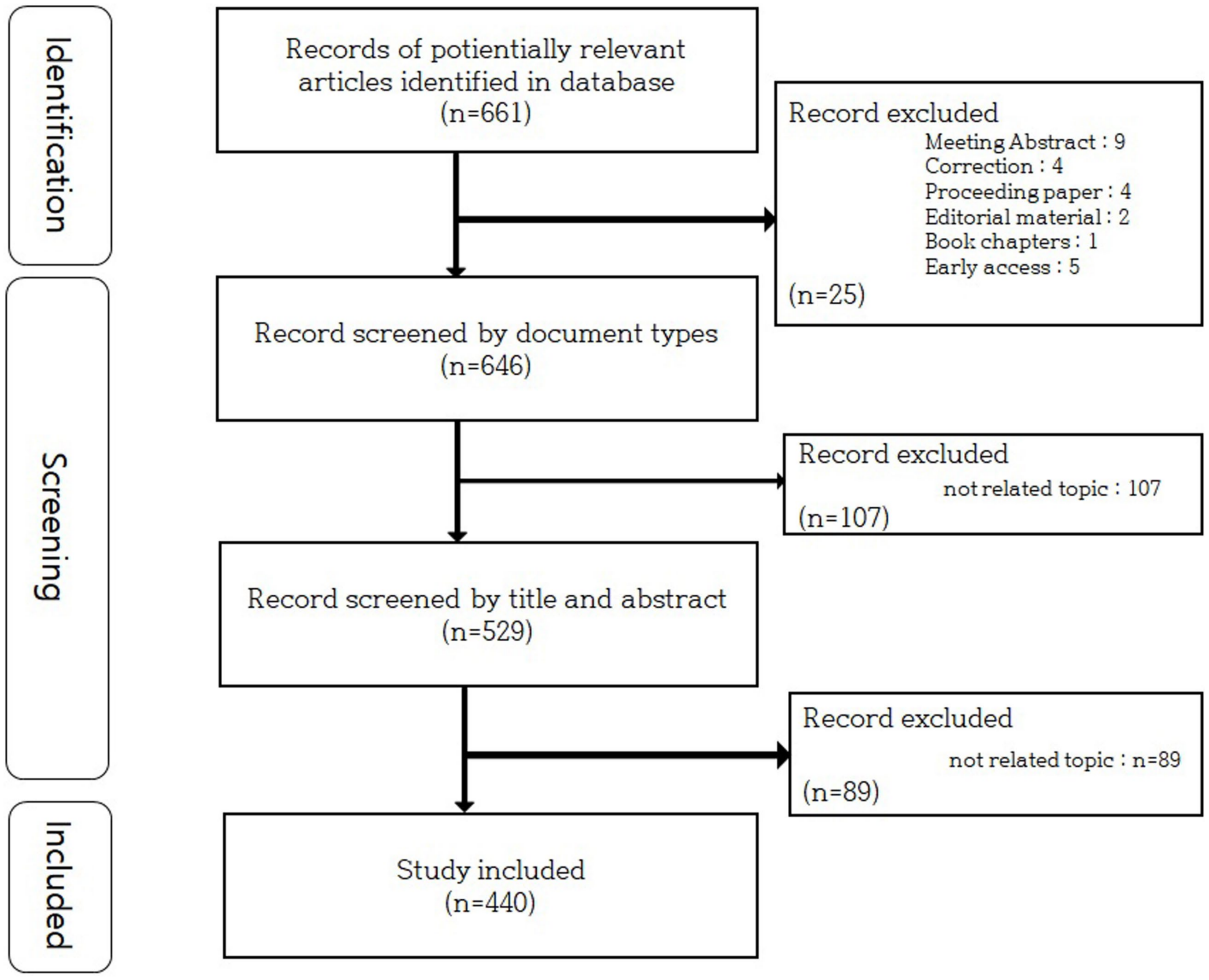
Flow chart detailing the process of article selection.

A total of 661 papers were extracted during the first data search. Articles and reviews were excluded, and 636 works of literature were selected. Two independent researchers screened the remaining papers by examining each title and abstract. Criteria of inclusion were: (1) Original article (2) Time range: published from 2003 to 2022 (3) research regarding EA as treatment for stroke. Criteria of exclusion were: (1) research regarding encephalopathy other than stroke (dementia, Alzheimer, etc.) (2) EA intervention without penetration of skin (laser acupuncture, transcutaneous electrical nerve stimulation, etc.) (3) article without abstract, book chapters, and editorial meetings (4) retracted or overlapped research. Papers with incomplete text were also excluded, while those with a full text were reviewed. In total, 440 papers were selected for this study (Figure 1).

Keywords were unified into a single form to enhance the accuracy of our analysis. Full names were used in the author analysis to enhance accuracy (Chen, LD To Chen, Lidian). The affiliation name was unified (fourth military medical university to air force medical university).

### 2.2. Data analysis

The screened publications were categorized and analyzed according to the publication year, nation, journal, research institution, author, and affiliation. Data on nation, affiliation, keywords, and authors were analyzed using VOSviewer to visualize the links between categories. Research areas were categorized using the WOSCC algorithm, which considers the characteristics of the journal. Each article was sorted into one to six research areas (Milojević, 2020). To visualize the analysis, layout attraction/repulsion values were adjusted, and settings were differentiated by category for clearer visualization.

## 3. Results

### 3.1. Published year

**Figure 2:**
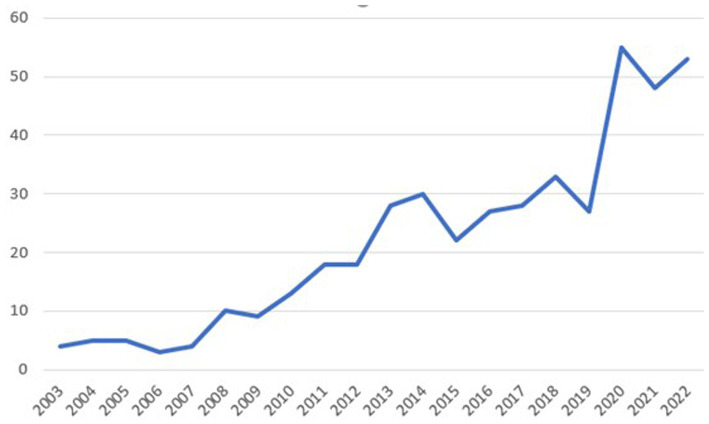
Annual trends of article publications.

In 2003, four papers were published, but in 2022, 53 papers were published. This result shows that the number of publications has increased from 27 in the year 2019 to 55 in 2020. The number of papers published doubled, and highest number of publications, 55, were in 2020, constituting 12.5% of all papers over 20 years (Figure 2).

### 3.2. Research field

Most papers were published in the field of neuroscience (36.6%), followed by Complementary Medicine (26.8%), Cell Biology (14.5%), and Medicine Research Experimental (14.31%) (Table 1). Among published papers, 294 papers were pre-clinical model and 45 papers were clinical trials. The remaining 101 researches were reviews.

**Table 1:** Distribution of publications on electroacupuncture for stroke by research fields.

Rank	Categories	Record count (*n*)	% (of 440)
1	Neurosciences	161	36.6
2	Integrative Complementary Medicine	118	26.8
3	Cell Biology	64	14.5
4	Medicine Research Experimental	63	14.3
5	Clinical Neurology	42	9.5
6	Medicine General Internal	36	8.2
7	Biochemistry Molecular Biology	20	4.5
8	Multidisciplinary Sciences	11	2.5
	Oncology	11	2.5
	Pharmacology Pharmacy	11	2.5
	Rehabilitation	11	2.5

### 3.3. Journals

Among the 440 papers, Neural Regeneration Research had the highest number of publications (10.9%). Evidence-Based Complementary and Alternative Medicine had the second most published papers (9.5%), followed by Acupuncture in Medicine (4.1%) and Medicine (3.2%) (Table 2).

**Table 2:** Journals with the most publications on electroacupuncture for stroke.

Rank	Journals	Record Count (*n*)	% (of 440)
1	Neural Regeneration Research	48	10.9
2	Evidence-Based Complementary and Alternative Medicine	42	9.5
3	Acupuncture in Medicine	18	4.1
4	Medicine	14	3.2
5	Journal of Traditional Chinese Medicine	12	2.7
	Neural Plasticity	12	2.7
7	Neurological Research	11	2.5
8	BMC Complementary Medicine and Therapies	9	2.0
	Brain Research	9	2.0
	International Journal of Molecular Medicine	9	2.0
	Trials	9	2.0

### 3.4. Countries

China published 374 papers, accounting for 85% of the 440 papers, and published the most number of research articles. The following nations were South Korea (8.4%), the United States (6.1%), and Taiwan (4.1%) (Table 3).

**Table 3:** Countries with the most publications on electroacupuncture for stroke.

Rank	Countries	Record count (*n*)	% (of 440)
1	Peoples R China	374	85.0
2	South Korea	37	8.4
3	USA	27	6.1
4	Taiwan	18	4.1
5	Australia	6	1.4
6	England	4	0.9
7	Canada	3	0.7
	Japan	3	0.7
9	Austria	2	0.5
	France	2	0.5
	Germany	2	0.5
	Singapore	2	0.5
	Thailand	2	0.5

**Figure 3:**
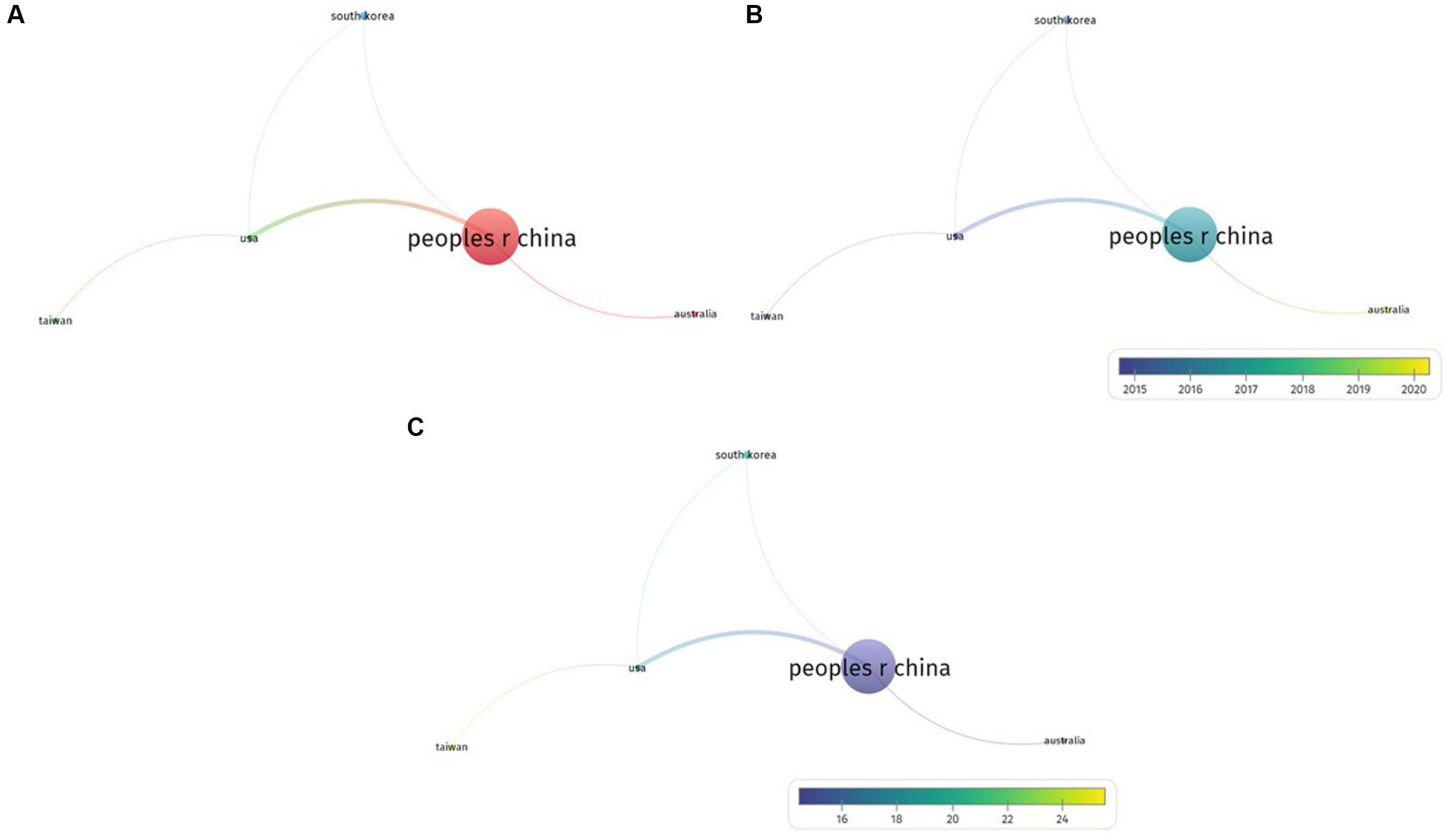
**(A)** A visualization of the network between countries which published on electroacupuncture for stroke. **(B)** A visualization of countries which published on electroacupuncture for stroke distinguished by average publication year. **(C)** A visualization of countries which published on electroacupuncture for stroke distinguished by average citations.

First, countries that published more than five papers were analyzed using the VOSviewer program, and three clusters were defined. The first cluster comprises China and Australia, the second cluster comprises USA and Taiwan, and third cluster includes South Korea (Figure 3A).

Subsequently, research papers from each country were distinguished based on the average publication year. As the average publication year increased, nodes were visualized in blue, and as they became more recent, they were visualized in red (Figure 3B).

Finally, the average citation count of the publications from each country was analyzed. Fewer cited country nodes were visualized in blue and more cited nodes were visualized in red (Figure 3C).

### 3.5. Affiliated institution

The analysis by affiliation showed that Fujian University of Traditional Chinese Medicine produced the highest number of studies (9.5%), followed by Guangzhou University of Chinese Medicine (7.7%), Air Force Military Medical University (5.9%), Tianjin University of Traditional Chinese Medicine (5.2%), and Fudan University (4.8%) (Table 4).

**Table 4:** Institutions and their number of publications.

Rank	Affiliations	Record count (*n*)	% (of 440)
1	Fujian University of Traditional Chinese Medicine	42	9.5
2	Guangzhou University of Chinese Medicine	34	7.7
3	Air Force Military Medical University	26	5.9
4	Tianjin University of Traditional Chinese Medicine	23	5.2
5	Fudan University	21	4.8
6	Shanghai University of Traditional Chinese Medicine	20	4.5
7	Nanjing University of Chinese Medicine	19	4.3
8	China Medical University Taiwan	17	3.9
9	Beijing University of Chinese Medicine	16	3.6
	Kyung Hee University	16	3.6

**Figure 4:**
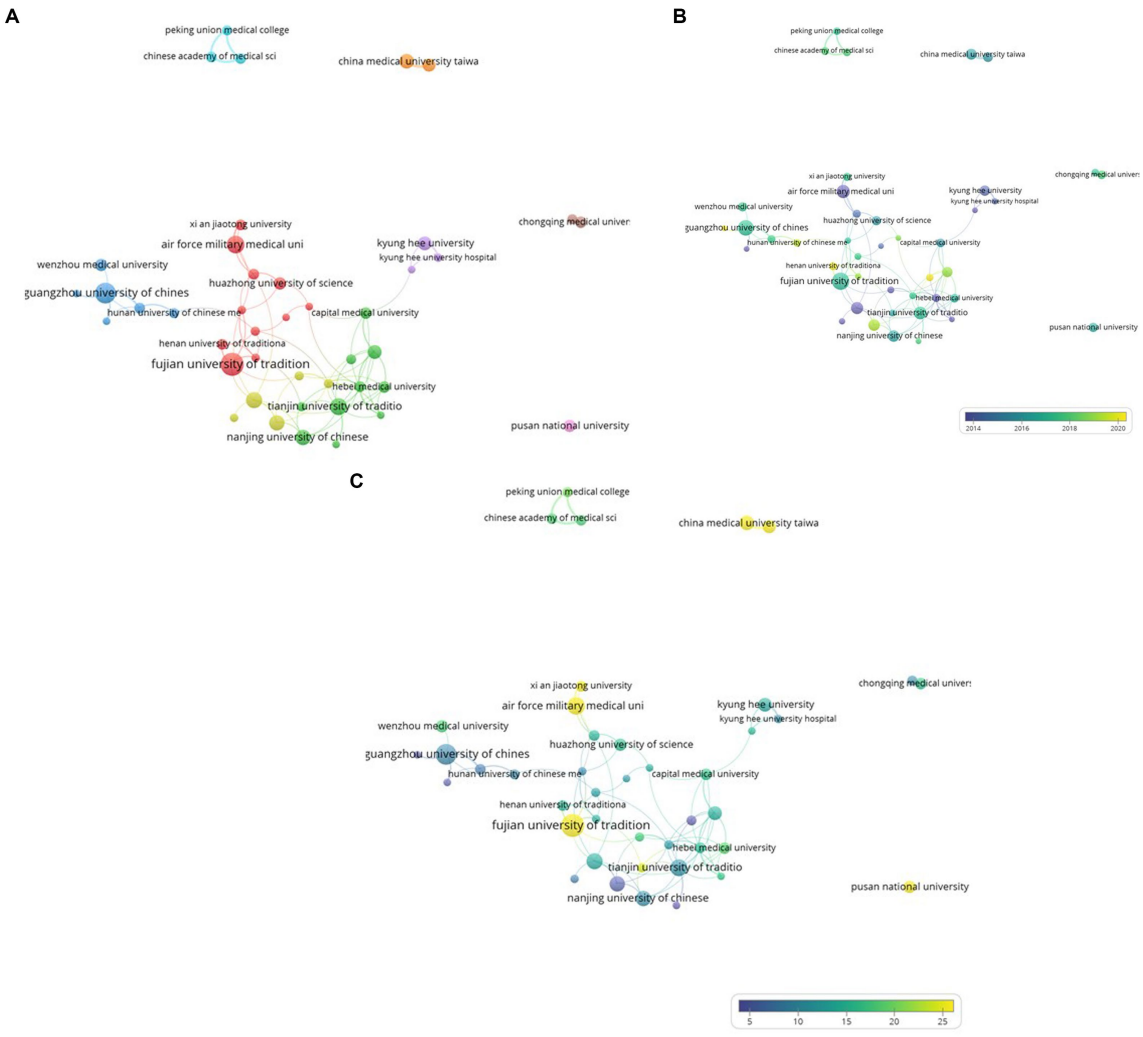
**(A)** A visualization of institutions which published on electroacupuncture for stroke. **(B)** A visualization of institutions which published on electroacupuncture for stroke by average publication year. **(C)** A visualization of institutions which published on electroacupuncture for stroke by average citations.

A total of 43 institutions published more than five research papers, which were categorized into nine clusters using the VOSviewer program (Figure 4A). Cluster 1 comprised 11 institutions, including the Fujian University of Traditional Chinese Medicine and the Air Force Military Medical University. In the second cluster, 10 institutions were included, comprising Tianjin University of Traditional Chinese Medicine and Nanjing University of Chinese Medicine. Cluster 3 comprised seven institutions, including Guangzhou University of Chinese Medicine and Wenzhou Medical University. Cluster 4 included five institutions, including Fudan University and Shanghai University of Traditional Chinese Medicine. Cluster 5 comprised three institutions: Kyung Hee University and Kyung Hee University Hospital. The Chinese Academy of Medical Sciences Peking Union Medical College, Peking Union Medical College, and Peking Union Medical College Hospital were included in cluster 6. Cluster 7 included the China Medical University Hospital Taiwan and China Medical University Taiwan. Cluster 8 included Chongqing Medical University and Southwest Medical University. Pusan National University alone was categorized as Cluster 9.

Second, the published research papers of each affiliation were distinguished based on the average publication year. As the average publication year increased, it was visualized in blue; if it became more recent, it was visualized in yellow (Figure 4B). Anhui University of Chinese Medicine had the most recent average publication year (2021.20), and The Guangxi University of Chinese Medicine had the oldest average publication year (2011.60).

Finally, the average number of citations of publications from each affiliation was analyzed. The more blue a node, the less frequently it is cited. The more yellow a node, the more frequently it was cited (Figure 4C). The least cited institution was the Heilongjiang University of Chinese Medicine (4.9) and the most cited institution was the Air Force Military Medical University (37.7).

Analysis by cluster showed that Cluster 5 had the earliest average publication year, and Cluster 8 had the latest. Cluster 9 had the largest average number of citations, and Cluster 3 was the least cited (Table 5).

**Table 5:** Analysis of institution cluster.

	Occurrence	Average publication year	Average citations
Cluster 1	12.5	2016.7	17.6
Cluster 2	8.9	2016.6	13.4
Cluster 3	12.3	2017.4	9.4
Cluster 4	12.2	2015.1	11.3
Cluster 5	9.0	2014.1	12.9
Cluster 6	9.3	2017.9	18.6
Cluster 7	15.5	2015.9	26.3
Cluster 8	9.5	2017.5	12.9
Cluster 9	13.0	2016.5	31.5

### 3.6. Author

The authors’ analysis revealed that Chen Lidian (7.7%) and Tao Jing (from Fujian University) published the most papers, followed by Liu Weilin (4.5%), Huang Jia (4.3%), and Xiong Lize (3.9%) from Tongji University (Table 6).

**Table 6:** Authors with the most publications on electroacupuncture for stroke.

Rank	Author	Record count (*n*)	% (of 440)
1	Chen Lidian	34	7.7
	Tao Jing	34	7.7
3	Liu Weilin	20	4.5
4	Huang Jia	19	4.3
5	Xiong Lize	17	3.9
6	Wang Qiang	16	3.6
7	Choi Byung-Tae	13	3.0
	Shin Hwa Kyoung	13	3.0
9	Lin Ruhui	12	2.7
	Xu Nenggui	12	2.7
	Yang Shanli	12	2.7

**Figure 5:**
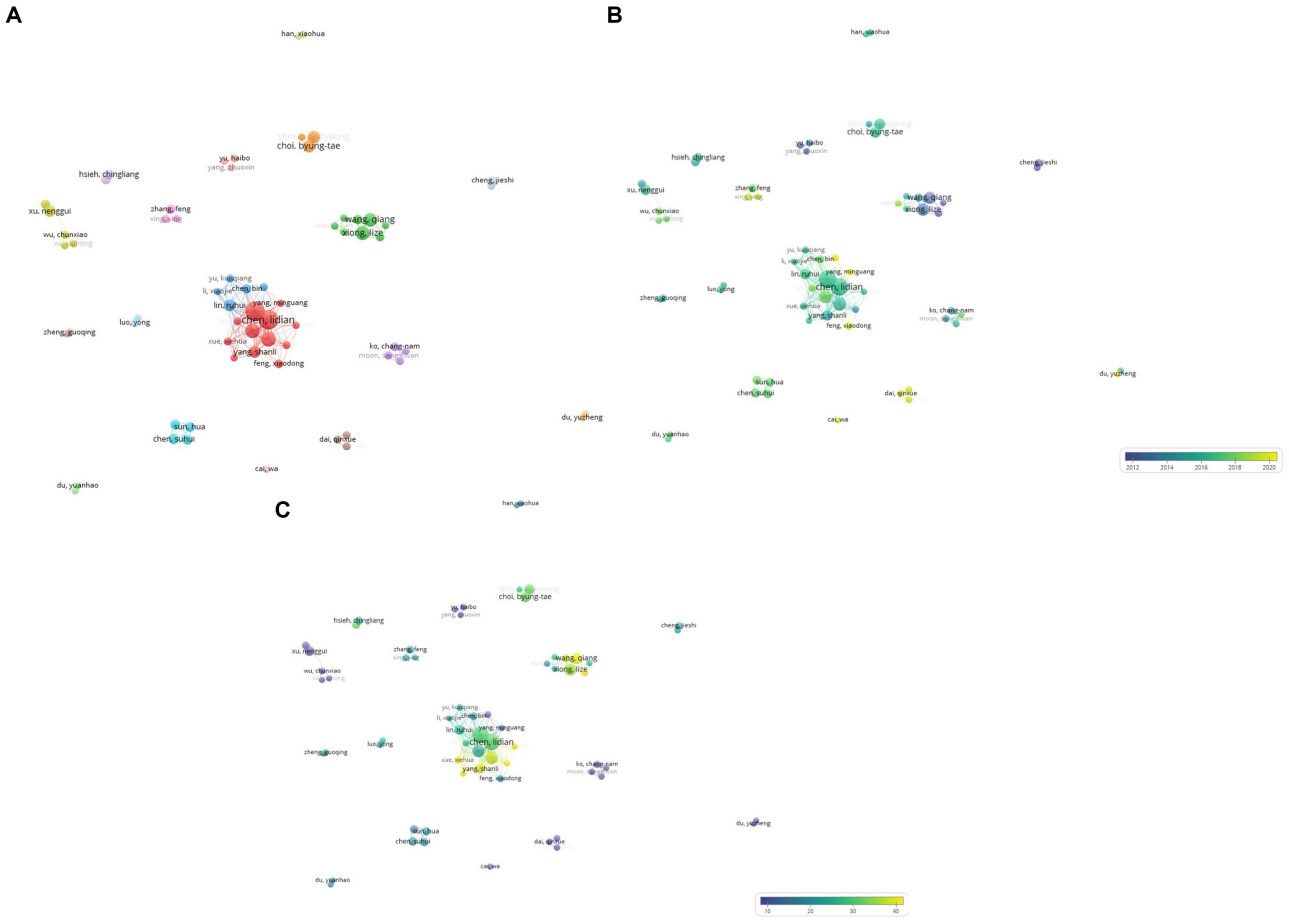
**(A)** A visualization of authors who published on electroacupuncture for stroke. **(B)** A visualization of authors who published on electroacupuncture for stroke by average publication year. **(C)** A visualization of authors who published on electroacupuncture for stroke by average citations.

First, those who published more than five papers (65 authors) were analyzed using the VOSviewer program, and 18 clusters were defined. Cluster 1 included 12 authors: Chen Lidian, Tao Jing, and etc. Eight authors, including Xiong Lize and Wang Qiang, were categorized in Cluster 2. Cluster 3 included five authors: Lin Ruhui, Chen Bin, and etc. In Cluster 4, there were five authors: Xu Nenggui, Yi Wei, and etc. Further clusters are visualized in Figure 5A.

The research papers published by each author were then distinguished by average publication year. As the average publication year increased, the authors cluster turned blue. The more recent the average publication year, the more yellow the author cluster (Figure 5B). Cheng and Jieshi had the oldest average publication year (2007.9), and Yang and Minguang had the most recent (2020.6).

Finally, the average number of publications by each author was determined. Blue indicates that the average number of citations is small. As the node color approached yellow, the average number of citations increased (Figure 5C). Chen and Shaoyang had the largest average citation value (57.7), whereas Du and Yuzheng had the smallest (1.4).

Analysis of each cluster showed that Cluster 12 had the earliest average publication year (2009.02) and Cluster 18 had the most recent average publication year (2019.06). Cluster 16 had the lowest average number of citations (3.9) and Cluster 7 had the highest average number of citations (32.62) (Table 7).

**Table 7:** Analysis of author cluster.

	Occurrence	Average publication year	Average citations
Cluster 1	13.3	2016.8	32.2
Cluster 2	8.5	2014.4	32.6
Cluster 3	7.4	2017.3	19.5
Cluster 4	7.2	2016.9	8.8
Cluster 5	6.0	2015.5	8.1
Cluster 6	9.3	2017.5	17.9
Cluster 7	10.3	2016.0	30.6
Cluster 8	6.0	2019.3	9.7
Cluster 9	6.3	2018.8	20.5
Cluster 10	5.3	2012.7	4.8
Cluster 11	5.0	2017.4	16.8
Cluster 12	6.5	2009.0	21.2
Cluster 13	5.0	2016.4	16.4
Cluster 14	9.0	2015.8	28.4
Cluster 15	6.0	2016.0	23.6
Cluster 16	5.0	2017.9	3.9
Cluster 17	5.5	2015.2	19.3
Cluster 18	5.0	2019.6	11.2

### 3.7. Keyword

**Figure 6:**
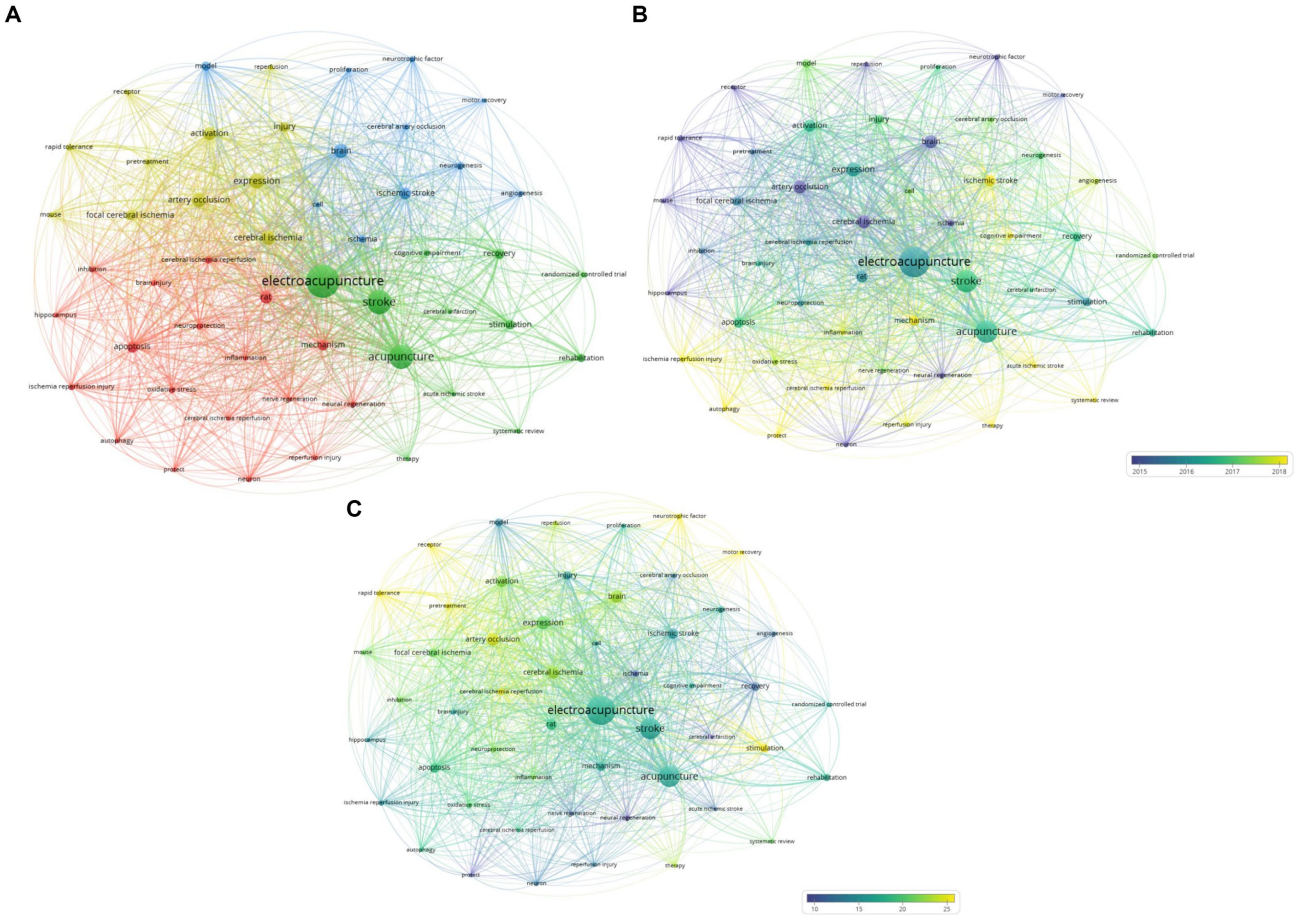
**(A)** A visualization of keywords related to electroacupuncture for stroke. **(B)** A visualization of keywords related to electroacupuncture for stroke by average publication year. **(C)** A visualization of keywords related to electroacupuncture for stroke by average citations.

First, 1,616 keywords were mentioned in 440 papers, and 52 keywords that were found more than 15 times were analyzed. Four keyword clusters were defined using the VOSviewer (Figure 6A).

The keywords were categorized according to the average publication year. As the average publication year increases, the results were visualized in blue. Because the average publication year was recent, it is visualized in yellow (Figure 6B). Analysis of keywords by average publication year showed that the three most recent keywords were “autophagy,” “systematic review,” and “cognitive impairment.” The three oldest keywords were “neural regeneration,” “cerebral ischemia,” and “arterial occlusion” (Table 8).

**Table 8:** The most frequent keywords related to electroacupuncture for stroke from 2003–2022.

Cluster	Keyword	Occurrence	Average publication year	Average citations
1	Rat	64	2015.9	18.7
	Apoptosis	58	2016.7	18.7
	Mechanism	47	2018.3	16.4
	Cerebral ischemia reperfusion	29	2016.0	24.7
	Neuroprotection	28	2015.9	21.5
	Oxidative stress	28	2017.5	19.8
	Ischemia–reperfusion injury	28	2017.9	14.9
	Brain injury	25	2016.4	15.7
	Inhibition	24	2015.5	21.3
	Neural regeneration	24	2013.7	8.5
	Neuron	22	2014.9	12.9
	Autophagy	19	2019.8	18.0
	Hippocampus	19	2014.8	15.2
	Inflammation	18	2017.8	21.4
	Reperfusion injury	17	2017.8	13.5
	Nerve regeneration	16	2017.2	11.6
	Cerebral ischemia–reperfusion injury	15	2018.4	18.1
	Protect	15	2018.5	10.3
2	Electroacupuncture	285	2016.0	17.2
	Acupuncture	178	2016.5	16.6
	Stroke	176	2016.8	16.5
	Recovery	49	2016.7	11.9
	Stimulation	45	2015.9	26.2
	Rehabilitation	36	2016.5	17.3
	Cognitive impairment	22	2019.1	16.9
	Randomized controlled trial	20	2017.3	16.4
	Systematic review	17	2019.2	20.7
	Cerebral infarction	16	2016.8	6.1
	Therapy	16	2018.3	22.7
	Acute ischemic stroke	15	2018.4	11.9
3	Brain	78	2015.1	22.4
	Ischemic stroke	60	2018.5	15.3
	Model	39	2017.2	14.0
	Ischemia	31	2014.4	11.4
	Neurogenesis	29	2017.0	16.4
	Neurotrophic factor	22	2014.9	25.8
	Cerebral artery occlusion	21	2017.3	13.3
	Proliferation	19	2016.7	17.0
	Cell	18	2017.3	15.5
	Angiogenesis	18	2017.6	12.8
	Motor recovery	15	2015.2	30.3
4	Expression	88	2016.2	20.7
	Cerebral ischemia	78	2013.8	22.2
	Artery occlusion	77	2013.9	24.0
	Activation	70	2016.6	21.6
	Focal cerebral ischemia	46	2015.7	20.7
	Injury	46	2017.0	15.2
	Rapid tolerance	27	2014.8	27.9
	Pretreatment	25	2015.5	30.0
	Mouse	20	2014.6	20.9
	Receptor	18	2014.7	24.8
	Reperfusion	17	2014.1	23.0

Finally, the keywords were sorted by the average number of citations. The smaller the average number of citations, the closer the color is to blue. The larger the average citation, the closer it is to yellow (Figure 6C). The results of keyword analysis by average citations showed that the three most-cited keywords were “motor recovery, pretreatment, and rapid tolerance” (see Table 8).

Comparison analysis between each keyword cluster showed that keywords from Cluster 4 had the oldest average publication year but were the most cited. Keywords from the second cluster were the most recent but were the least cited (Table 9).

**Table 9:** Analysis of keyword clusters.

	Occurrence	Average publication year	Average citations
Cluster 1	27.6	2016.8	16.7
Cluster 2	68.6	2017.3	16.4
Cluster 3	31.8	2016.5	17.7
Cluster 4	46.5	2015.2	22.8

## 4. Discussion

To conduct a bibliometric analysis of EA in patients with stroke, 440 papers published over the last 20 years were reviewed. The number of publications has gradually increased annually. In the first 10 years, starting from 2003, 89 papers were published, and in the last 10 years, 351 papers were published. By 2020, more than 45 papers had been published annually. We believe that this is because EA has been extensively studied and is actively used clinically to treat stroke.

The field of Neurosciences had the most research, followed by the fields of Integrative Complementary Medicine, and Cell Biology. In the field of Neuroscience, research focusing on this mechanism has been actively conducted because of objective and quantitative measurements of the frequency and strength of EA stimulation (Napadow et al., 2005).

Neural Regeneration Research journal had the most numerous publications, followed by Evidence-Based Complementary and Alternative Medicine, and Acupuncture in Medicine. The multiplication of studies in neuroscience is reflected by the published journals. EA treatment is a complementary medical method that has been actively studied.

China published the highest number of studies, followed by Korea and the United StatesIt appears that this is proportionally related to the fact that China is the center of worldwide TCM research. Analysis of clusters showed China and Australia, which recently conducted research in this field actively, was included in the same cluster. Australia conducted studies on the use of EA for the sequelae of stroke. In the early stages, the United States and Taiwan conducted highly cited and influential studies. United States has the highest medical expenditure of post stroke care per patient in the world (Rajsic et al., 2019), and in order to lower this social cost, complementary medicine has been actively researched. In the United States, there was early interest in the combination of Western and Eastern rehabilitation techniques to improve the effectiveness of rehabilitation treatment (Alexander et al., 2004). Appropriate intensity and frequency of EA intervention has been reported to show neuroprotection by up-regulation of the blood flow speed in the infarct site (Zhou et al., 2011) and meta-analysis of acupuncture combined with a conventional rehabilitation approach suggested acupuncture provided additional benefit to motor recovery compared to conventional rehabilitation approach alone. However, some clinical studies failed to demonstrate treatment effect of acupuncture/EA on stroke and inconsistency between studies about effect of EA on stroke remains major obstacle to actively apply EA in rehabilitation program for stroke. This may be the reason why research was actively conducted in the United States in the beginning but has been sluggish recently. Although researches on EA have been increased worldwide, international co-operation width and link strength of collaboration between nations were weak. One of the reasons for the lack of international collaboration might be differences in the frequency of use of acupuncture in clinical settings and controversies over the underlying mechanism of acupuncture. In order to facilitate the growth of the field and sustain high-quality work, researchers need to give effort to multi-national interaction and co-operation in the future. By affiliated institution, Fujian University of Traditional Chinese Medicine published the largest number of studies, followed by Guangzhou University of Chinese Medicine and Air Force Military Medical University. Chen Lidian and Tan Jing published the most papers, followed by Liu Weilin. The three most published authors were affiliated with Fujian University of Traditional Chinese Medicine and were included in author cluster 1, studying mainly the neuroprotective mechanism and anti-inflammatory effects (Lan et al., 2013) of EA for stroke through animal model experiments. By analyzing the top authors, researchers from the same institution cooperated but lacked collaboration with other institutions, nations, or authors. Additional network composition is needed in the future to increase academic status and diversity.

Keyword analysis was performed to identify the research trends and contents of the published papers. The three oldest keywords by average publication year were “neural regeneration, cerebral ischemia, and artery occlusion.” In a previous study, an animal model of ischemic stroke created through the middle cerebral artery and internal carotid artery occlusion and reperfusion was used to verify the nerve regeneration effect of EA (Ma et al., 2020). The three most recent keywords were “autophagy, systematic review, and cognitive impairment.” Studies on the neuroprotective and neuroplastic regulatory effects of EA are continuously in progress, and the autophagic effect of EA-related signal transduction pathway regulation is a recent research topic receiving attention. In clinical research, studies on the sequelae of stroke, such as cognitive disorders (Xie et al., 2022), depression (Wang et al., 2021), and rigidity (Cai et al., 2017) are actively being conducted.

The published research keyword clusters were categorized according to “neuroprotection,” “clinical rehabilitation,” “neuroplasticity,” and “pretreatment-induced tolerance.” The first cluster included “rat,” “apoptosis,” and “mechanism” and was categorized as a “neuroprotection” topic. Neuroprotection focuses on minimizing additional damage of nerve cells after acute brain injury (Chamorro et al., 2021) and EA showed neuroprotective effects against acute brain damage by inducing cell apoptosis, anti-inflammation, and autophagocytosis. As our understandings of the mechanisms and pathology of stroke increases, neuroprotective strategies emerged as hopeful treatment since acute stroke in followed by secondary neuroinflammation and recent studies report neuroinflammation as a key determinant of stroke prognosis (Tao et al., 2020). Nonetheless, there has been no neuroprotective drugs with proven clinical efficacy. EA could display a promising neuroprotective property in stroke individual but additional studies are needed to further elucidate and validate its mechanism and effectiveness. The second cluster consisted of “recovery,” “stimulation,” “rehabilitation,” and “cognitive impairment” and the topic was categorized as “clinical rehabilitation.” Timely rehabilitation treatment from the acute stage of stroke seems to lower risk of physical disability and complication. Also, recent clinical research showed that enhanced functional recovery is possible even in the chronic stage of stroke after 6 months (Zhao and Willing, 2018). Stroke sequelae include neurological and neuropsychological deficits. A human clinical study reported EA showed improvement of functional impairment of sequelae such as dysphagia (Huang et al., 2020) and aphasia (Shi et al., 2022) and improved neuropsychiatric sequelae following stroke, such as cognitive impairment and post-stroke depression (Wang et al., 2021). In the third cluster, “neurogenesis,” “neurotrophic factor,” “proliferation,” and “angiogenesis” was included, and the topic was categorized as “neuroplasticity.” In the sub-acute and chronic stroke brain, voluntary healing occurs and this non-cure healing process happens through brain plasticity (Zhao and Willing, 2018). Neurogenesis, angiogenesis, and synaptic regeneration are important for voluntary neuroplasticity in the brain. Neuroplasticity modifies functional activity by learning through responses to stimulation and plays an important role in functional recovery when impairment occurs because of incomplete nerve cell regeneration. In animal experiments, EA showed regulatory effects on neuroplasticity by accelerating neurogenesis, inducing angiogenesis, and regulating neuroglial cells, such as astrocytes, oligodendrocytes, microglial cells, and neurotrophic factors (Zhang et al., 2021). In the fourth cluster, “expression,” “activation,” “injury,” “rapid tolerance,” and “pretreatment” keywords were included, and the topic was categorized as “pretreatment induced tolerance.” EA pretreatment controls the cannabinoid system, oxidative stress, and growth factors and suppresses cell apoptosis, resulting in neuroprotective effects (Li et al., 2012). Therefore, EA pretreatment can be used as a preventive and initial treatment strategy for patients at risk of ischemic stroke, and many studies have been conducted to prove this neuroprotective effect (Wang et al., 2009). The research included in Cluster 4 focused mainly on verifying the stroke prevention and early neuroprotective mechanisms of EA pretreatment, and there have been almost no clinical studies to date.

Comparing through average publish year, cluster 4 “EA pretreatment induced rapid tolerance” studies were conducted in the past than the other clusters and also showed high average citation. Cluster 2, a “clinical rehabilitation study,” had a more recent average publication year, and the average citation rate was lower than that of the other clusters. Overall, animal model studies have mainly been conducted in the past, and we found that human-subject clinical studies have recently increased in animal experiments.

This study has several limitations. First, only the WOSCC was used as the extraction source for the research papers. WOSCC was used in this study because the database provides high quality and globally influential research papers and details of the research papers needed for bibliometric analysis. If other databases from East Asian countries that were actively studying the field were included as research sources, the results would have been more significant, but this study focus on database which is used worldwide. Second, the number and name of each cluster were discussed between two individual researchers and defined because individual subjectivity can intervene in the process of defining its properties, hindering objectivity; however, researchers may have different views. Considering the research trend on EA about stroke, mechanism of EA on neuroplasticity and its consequent motor recovery might be of interest of future research. Despite major advances in understanding pathophysiology and acute phase stroke intervention, not much progress has been made especially in chronic phase. Because timely intervention is crucial in stroke rehabilitation, EA can be a relatively convenient and cost-efficient intervention especially where Conventionally therapy is not readily available. To achieve this, large scale, high-quality, multicenter or multinational clinical trials must be further conducted to adequately measure the proper effect size. Additionally, analysis of the research trends in various TCM treatment methods, such as manual acupuncture, herbal medicine, and pharmacopucture for stroke could be conducted to provide insight into stroke treatment and rehabilitation.

## 5. Conclusion

Using WOSCC as a source, the research trends of 440 papers on EA as a treatment for stroke were analyzed using a bibliometric method, and the results were as follows:

1. In the last 20 years, published studies have gradually increased, and especially between 2019 and 2020, the number has more than doubled, indicating that research has been actively conducted until recently.
2. Studies have been conducted in the fields of neuroscience and complementary medicine.
3. China had the largest number of publications, followed by the United States and other East Asian countries, such as South Korea and Taiwan.
4. Fujian University of Traditional Chinese Medicine published the most research papers, whereas Chen Lidian and Tan Jing published the largest number of studies.
5. The keyword analysis indicated topics related to “neuroprotection,” “clinical rehabilitation,” “neuroplasticity,” and “pretreatment-induced tolerance.”

## Data availability statement

The original contributions presented in the study are included in the article/supplementary material, further inquiries can be directed to the corresponding author.

## Author contributions

HC: Conceptualization, Data curation, Formal analysis, Investigation, Writing – original draft. W-CS: Resources, Writing – review & editing. J-mK: Investigation, Writing – original draft. HK: Methodology, Software, Writing – review & editing. J-HC: Supervision, Validation, Writing – review & editing. M-YS: Supervision, Validation, Writing – review & editing. W-SC: Conceptualization, Project administration, Supervision, Writing – review & editing.
